# Fully Automated Algorithm for Light Pole Detection and Mapping in Rural Highway Environment Using Mobile Light Detection and Ranging Point Clouds

**DOI:** 10.1177/03611981221082531

**Published:** 2022-03-17

**Authors:** Maged Gouda, Amr Shalkamy, Xinyi Li, Karim El-Basyouny

**Affiliations:** 1 Department of Civil and Environmental Engineering, University of Alberta, Edmonton, AB, Canada; 2 State Key Laboratory of Information Engineering in Surveying, Mapping and Remote Sensing, Wuhan University, Wuhan, China

**Keywords:** data and data science, information systems and technology, big data analytics, data analytics, remote sensing, infrastructure, roadway design, low-volume roads, asset management, safety, roadside safety design, point cloud data, LiDAR data, light poles mapping

## Abstract

The widespread use of light detection and ranging (LiDAR) data provides a promising source for the automatic detection and inventory of roadside assets. One of the essential elements in roadside furniture is light poles. There is limited research on the mapping of light poles using point-cloud data on rural highways. In this environment, the placement of light poles within roadside clear zones often poses a safety concern, as they are related to an increased risk of collisions. Only a limited number of studies have explored the relationship between light poles and safety because of the time-consuming and labor-intensive practices of collecting light pole assets data using traditional manual methods. This paper proposes an automated approach to mapping the locations of light poles. First, the scanning vehicle trajectory is extracted, smoothed, and then used to segment the point-cloud data into smaller overlapped batches of data. Several filters are applied to extract pole-like objects from the data. The segments are combined back together, and a density-based clustering algorithm is used to group the remaining points into clusters. A geometric filter is finally applied to extract light poles. The model is tested on 28 km of data on three rural highways in Alberta, Canada. The proposed algorithm is found to be accurate relative to previous studies, with average precision, recall, and F1 scores exceeding 98% for the test segments. The proposed work can assist in the automation of light pole inventory and road safety audits by transportation agencies.

Road furniture such as traffic signs, safety barriers, fences, and lighting poles is crucial in facilitating efficient and safe travel on highways. Roadside safety has been a major concern for highway designers, with a significant part of this concern relating to crashes involving utility poles. Because of the high structural strength of utility poles and the possible damage they could cause, pole-related collisions tend to be more severe (*1*). According to the Fatality Analysis Reporting System (FARS) (*2*), in 2018, there were 684 fatal collisions related to utility poles in the United States. Because of the severity of this type of crash, there is a need to collect information on locations and lateral offsets of lighting poles to ensure acceptable placement of these features from the highway travel edge. Thus, an accurate inventory of roadside features, including light poles, is increasingly demanded for various purposes such as safety audits, maintenance operations, and asset management. The availability of such information could help designers provide a safer and more recoverable roadside environment (*1*).

Information on roadways and roadside features can be collected using different approaches, including traditional surveying methods or emerging technologies such as remote sensing techniques. Obtaining information on road features such as utility poles using the conventional methods, especially on a large scale, is time-consuming, costly, and potentially infeasible. This is because of the hurdles associated with manual inspections, such as the massive workload required, long site visits, human errors, and the difficulty in conducting such data collection periodically (*3, 4*). With the evolving use of the three-dimensional (3D) models of highway corridors, there has been a wide range of applications to collect information on road geometry and furniture and assess the safety of these facilities (*5–10*). Surveying, photogrammetry, and remote sensing have been commonly used, but the emerging popularity of light detection and ranging (LiDAR) data has shifted the attention to utilizing LiDAR point clouds in the detection and mapping of road features, including lighting poles (*11*).

LiDAR is a technology that integrates laser scanners, global navigation satellite systems (GNSS), and inertial measurement units (IMU) into one scanning system to collect point clouds of the roadway environment. These point clouds can be highly dense, which depends on the field of view of the scanner, the speed of the vehicle the scanner is attached to, and the scanning rate. By having heavily dense point clouds, the resolution will be higher at the cost of increasing the data set size and the processing speed (*12*). LiDAR is most commonly used in systems such as airborne laser scanning (ALS), terrestrial laser scanning (TLS), and mobile laser scanning (MLS). In MLS, the scanning system is mounted on a data collection truck traveling at posted speed limits, collecting 360° virtual scans of highway segments consisting of highly dense point clouds (*13, 14*).

LiDAR data are associated with several benefits. The high density of mobile LiDAR data has enabled the efficient and accurate mapping of various highway features (*4, 15*). Also, using LiDAR data greatly enhances the safety of the process of collecting information on highways as a result of eliminating the presence of surveyors next to the traffic stream. Moreover, the spatial information about road features can be directly extracted from the georeferenced laser scanner data, minimizing the manual work and thus improving the efficiency and eliminating human error (*16, 17*).

Although there has been some progress in extracting light poles using mobile LiDAR data, previous studies centered on identifying and mapping lighting poles in urban environments using short and relatively flat roadway segments. However, the detection and mapping of light poles on rural highways are lacking research. The main differences between urban and rural environments for extracting such information are related to terrain and speed limit. First, urban roads are known to have flat terrain compared with the rolling to mountainous nature of rural highways along with the high variation in their vertical alignment in most cases (*14*). When extracting utility poles from LiDAR data segments located in rolling or mountainous terrain, separation of non-ground and ground objects, which is a major step in the algorithms developed in previous studies, would be challenging. Second, the speed at which LiDAR scans are conducted affects the properties of collected data, such as point density, based on which the extraction methodology must be different (*18, 19*). Also, since speed is one of the main factors affecting crash severity, pole-related collisions are expected to be more severe on rural highways than on urban roads. Therefore, there is a need for a robust method to collect information about lighting poles on rural highways.

This paper aims to introduce a fully automated approach for the identification and extraction of light poles in a rural setting in an efficient manner enabling the feasibility of large-scale implementation. The proposed method involves LiDAR data preprocessing, applying several filtration methods to extract pole-like candidates, clustering, detection, and mapping of poles. To validate the performance of the proposed method and ensure its applicability on a large scale, the algorithm was tested using LiDAR data of 28 km of highway segments in Alberta, Canada. The method validation revealed that the developed algorithm is accurate and precise, and outperforms currently available tools. The proposed method represents a tool that could help transportation agencies in roadside assessments and safety audits associated with light poles. It can also be used to maintain an updated inventory of information on these features. Moreover, this information could be useful in street management and visibility studies.

## Previous Studies

There have been a fair number of studies focused on the detection of light poles using LiDAR point clouds, with a particular emphasis on urban roads. One of the early studies was conducted by Lehtomäki et al. (*20*) to detect pole-like objects using mobile LiDAR data of urban streets. They used a scan-line-based algorithm to look for poles in sweeps (i.e., groups) of points in each scan line in LiDAR data. The authors reported a detection rate of 77.7% with false-positive detections, including pillars and building structures. In a different study, Yokoyama et al. (*21*) proposed a method to recognize pole-like objects from MLS point clouds utilizing a *k*-nearest neighbors graph and principal component analysis (PCA). The authors recommended future research to classify pole-like objects into light poles and traffic signs.

Golovinskiy et al. (*22*) presented a density-based methodology to recognize features of urban roads, including poles. The method was based on locating and processing clusters of 3D points according to their spatial context and configuration. Labeled data from a training data set were used to classify the identified features. The authors reported a recognition rate of 65%. Pu et al. (*23*) introduced a method for recognizing pole-like objects from MLS data. They used different shape and geometric attributes to detect these features. The authors reported a success rate of 86%. Yan et al. (*18*) utilized LiDAR data of an urban site in Ontario, Canada, to propose a method by which poles and towers can be detected. The procedure included ground filtering based on the statistical distribution of points, clustering based on point density, classification utilizing various decision rules, and data cleaning using a least-square circle fitting. The detection rate of the proposed method was 91%. Yu et al. (*24*) introduced a semi-automatic method for light pole extraction from mobile LiDAR data in an urban environment. The detection process consisted of road surface segmentation into road and non-road points followed by light pole extraction. The validation results of the proposed method showed a correctness rate of over 97%.

Zheng et al. (*25*) extracted pole-like objects from mobile LiDAR data. The authors introduced vegetation removal and pole detection methods. The proposed algorithm was tested on airborne and mobile LiDAR data. The authors concluded that the proposed method is accurate in both vegetation removal and pole detection.

Another urban-focused pole-like object detection method was proposed by Yang et al. (*26*), wherein poles are automatically detected after a four-stage process. First, building facades are filtered out to reduce the risk of false positives. The filtered point cloud is then subjected to a slice-based Euclidean clustering algorithm to establish pole-like candidate objects. A PCA shape recognition method is used to further filter the candidates as either pole-like or non-pole-like. With the locations of the pole-like objects known, a Voronoi constrained vertical region growing algorithm is used to fully realize the poles in the 3D point cloud. Results yielded an extraction quality of 90.43%, a recall value in the range of 92.61% to 94.56%, and a precision of 95.17% to 96.99%. While processing time was not discussed in the study, the authors acknowledge that work needs to be done on the method’s computational efficiency.

Some studies used voxel-based methodologies to detect and extract roadside features. Focusing on the detection of trees in an urban environment, Wu et al. (*27*) developed a voxel-based method to detect street trees from LiDAR data. Utilizing voxels and searching neighborhoods, the method focused on extracting potential trees and using morphological parameters to eliminate pole-like objects that are not trees. The method was tested on two, roughly 300 m long, segments of urban streets with a maximum of 1 m variation in their vertical profile. The results showed that the correctness of the detection rates exceeded 98%.

Tu et al. (*28*) developed a two-stage urban pole-like object extraction method based on plane filtering. The first stage involves fitting voxels to planes via an octree-based split scheme. After the plane fitting, the authors use plane filtering to extract pole-like objects based on either innate geometric features (the planar orientation, planar shape feature, and planar width) or isolation relative to the points surrounding the pole candidate. The completeness, correctness, and quality of this approach can reach up to 87.66%, 88.81%, and 79.03%, respectively.

Lehtomäki et al. (*29*) proposed a voxel-based method to map road environment infrastructure using MLS point clouds of urban streets. The stages of the extraction procedure included separating ground objects, segmentation, classification, and object location estimation. The authors successfully classified several objects, including road signs, pedestrians, vehicles, and posts. The method was tested on a 900 m road segment with a reported recall of 49.8%, 80.5%, and 66.0% for trees, lamp posts, and traffic poles respectively. Cabo et al. (*11*) used voxels to develop a method to automatically extract pole-like objects from mobile LiDAR data of urban streets. The method involved simplifying the LiDAR point cloud using voxels, analyzing and segmenting the layers of the voxelated point cloud horizontally, and implementing two-dimensional analysis to identify pole-like objects. The algorithm was tested on four sites and was able to detect pole-like objects with an average correctness rate of 83.8%, and a completeness rate of 92.3%.

Wu et al. (*30*) provided a supervoxel-based approach to extract street light poles in mobile LiDAR point clouds of urban streets. A RIEGL VMX-450 system was used to collect three highly dense data sets at low speeds of 40–50 km/h in Xiamen, China. The method consisted of five steps: preprocessing, localization, segmentation, feature extraction, and classification. The processing time required between ground filtering and labeling of light poles on 78 million, 220 million, and 403 million point clouds is 404.9, 1,032.5, and 1,806.6 s, respectively. The algorithm was tested on three data sets to detect light poles with an average recall, precision, and F1-score of 98.8%, 99.4%, and 98.6%. Two limitations discussed by the authors are the chances of over-segmentation when a light pole is located near heavy vegetation and the possibility of incomplete pole data. However, these two limitations do not affect the performance in finding light pole locations.

Guan et al. (*31*) proposed a method to detect pole-like objects from LiDAR data of an urban road area. A RIEGL VMX-450 system was used to survey an 11 km segment of the Ring Road South in Xiamen, China, at an average speed of 30–40 km/h. The scanning achieved a point-cloud density of 4,082 points per square meter (points/m^2^). The method consists of two stages: training and object detection. The training stage creates a contextual visual vocabulary via supervoxel segmentation to depict features of pole-like objects. The detection stage converts each type of object from the contextual visual vocabulary into a “bag-of-contextual-visual-words” representation, which is then compared against pole-like object candidates to detect similarities. The correctness, omission, and commission achieved are 88.9%, 11.1%, and 2.8%, respectively. The processing was performed on an 8-core-16-thread workstation, and the total processing time was 81 min (4,860 s).

An automated method for the localization and classification of pole-like objects along a semi-urban expressway was proposed by Ha et al. (*32*). The study applied this method to two MLS point clouds gathered from an urban road. After points of noninterest were eliminated from the point cloud, Euclidean clustering was used to group points into distinct clusters. The points were then voxelized, and the voxels forming pole-like objects were selected using a vertical height analysis. For classification, the pole-like objects were separated into nine different types based on their traffic functionalities and geometric shapes. The method was tested on two expressway segments, with an average recall of 92.9%, precision of 95.8%, and F1-score of 94.3%. The authors noted that limitations to the method could be overcome with additional training data.

With the lack of studies on pole detection on rural highways, a recent study by Gargoum et al. (*19*) proposed an algorithm for detecting light poles on rural highways using mobile LiDAR data. The method involved data tiling and voxelization, ground and non-ground filtering, and pole detection. The method was tested on a 4 km segment with a reported detection precision, recall, and F1-score of 68%, 49%, and 56.96%, respectively. The authors applied a similar method to those used in research on pole extraction in urban environments. The authors attributed significant misclassifications to the significantly low point density on rural roads as scanning is performed at high speeds of 90–100 km/h. Occlusion by other objects also affected the performance of the proposed method. Moreover, the rolling terrain on highways makes the extraction of non-ground objects, while maintaining pole points, a challenging task. Non-ground point extraction is integral to most pole extraction methods in the literature. The authors suggested that a more efficient and robust method is required to detect light poles in low-density point clouds collected at high speeds.

Another recent study by Yan et al. (*33*) proposed a workflow to detect and classify pole-like road objects using LiDAR data in a highway environment. The workflow consists of data preprocessing, detection, and classification. First, the ground points are removed by a ground filtering algorithm, and an iterative minimum-cut (min-cut) based segmentation approach is used to segment overlapping segments. More filters are then applied to detect pole-like objects, which are then classified using a random forest classifier. The method was tested on two highway data sets, with a reported recall of 94.9% and 97.8%, and precision of 92.7% and 94.1%. The authors discussed several limitations, such as the possibility of incomplete street lamp detection. For the two data sets, with 180 million and 316 million points, the processing times reported for the steps between ground removal and detection (without classification) were 7,235.1 and 12,570.8 s, respectively.

As evident from the review, most previous studies focused on pole recognition and mapping on urban streets, with less attention given to rural highways. Urban LiDAR data are typically collected at low speeds and provide an extremely dense point cloud and a high-resolution representation of the environment (*18, 19*). A voxel-based method that followed a similar approach to those used on urban data showed significantly lower performance on low-density rural point clouds collected at high speeds (*19*). Another study by Yan et al. (*33*) showed improved performance at the expense of significantly larger processing time. The studies conducted in rural environments indicated lower recall and precision rates and large processing times, showing the need for improvement. Thus, a more robust methodology for light pole detection on rural highways is required. This paper introduces a fully automated methodology for the detection and extraction of light poles. The proposed method operates directly on point-cloud points without the need to transform the data to volumetric representations (e.g., voxels), which yields unnecessarily expanded data, requires large processing time, and affects existing features associated with the original point (*30, 34*, *35*). The proposed algorithm is a powerful tool that can be used by transportation agencies to collect and maintain an updated database of information on light poles along rural highway networks.

## Data Collection

**Figure 1.**
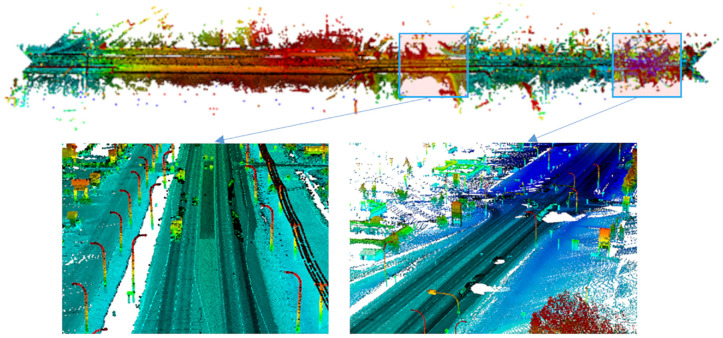
Example of point-cloud data collected on Highway 1.

LiDAR data collection was performed by Alberta Transportation in 2015 using a Tetra Tech PSP-7000, a proprietary multifunction pavement surface profiling vehicle. The vehicle had a RIEGL VMX-450 system to collect 360° LiDAR point clouds on rural highways in the province of Alberta, Canada. Surveys were conducted in normal traffic flow at speeds of up to 100 km/h. Collected data for a given highway are saved in 4 km segments in separate LAS files, each around 500 MB. Surveys performed at 90 km/h generate point densities in the range of 150 and 1,000 points/m^2^. In this study, data were collected on five 4 km segments on Highway 1, one 4 km segment on Highway 20, and one 4 km segment on Highway 11A in Alberta, Canada. An example of point-cloud data collected on Highway 1 is shown in Figure 1.

### Test Segments

**Figure 2.**
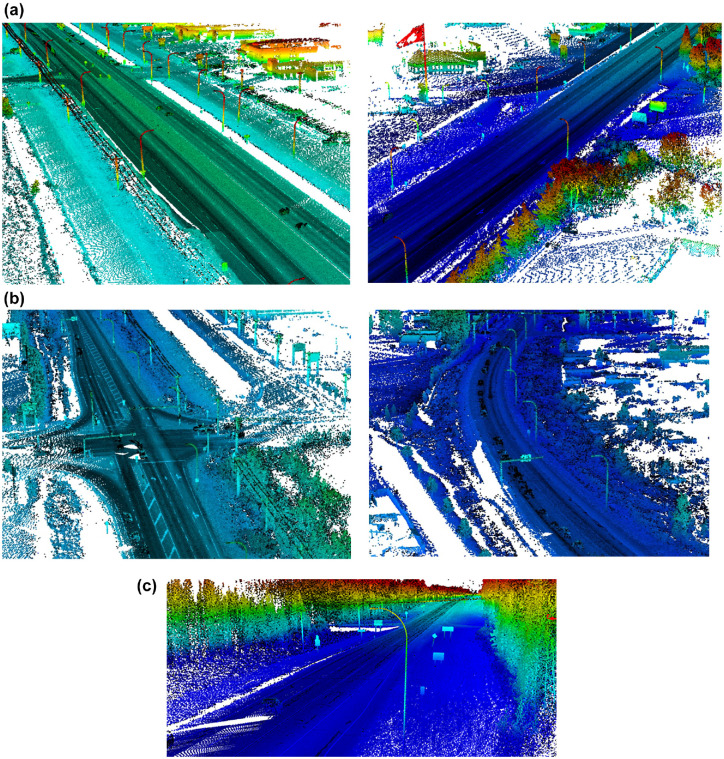
Collected data on rural highways in Alberta: (*a*) Highway 1, (*b*) Highway 20, and (*c*) Highway 11A.

- Highway 1: Five 4 km segments from this highway were used for analysis (Figure 2*a*). The segments are from a multi-lane highway located about 50 km to the east of the City of Calgary. These segments had many light poles.
- Highway 20: A 4 km segment from Highway 20 was used for analysis (Figure 2*b*). This segment is a two-way-two-lane road located in Sylvan Lake, about 20 km to the west of the city of Red Deer.
- Highway 11A: A 4 km segment from Highway 11A was used for analysis (Figure 2*c*). This segment is on a two-way-two-lane road located 7 km northwest of the city of Rocky Mountain House.

## Methodology

**Figure 3.**
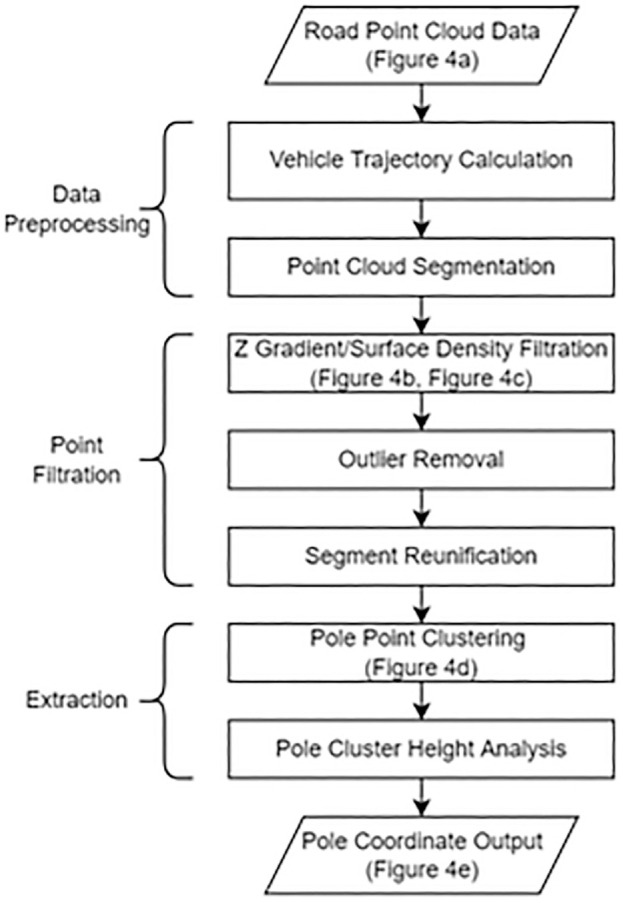
Flowchart of the proposed methodology.

**Figure 4.**
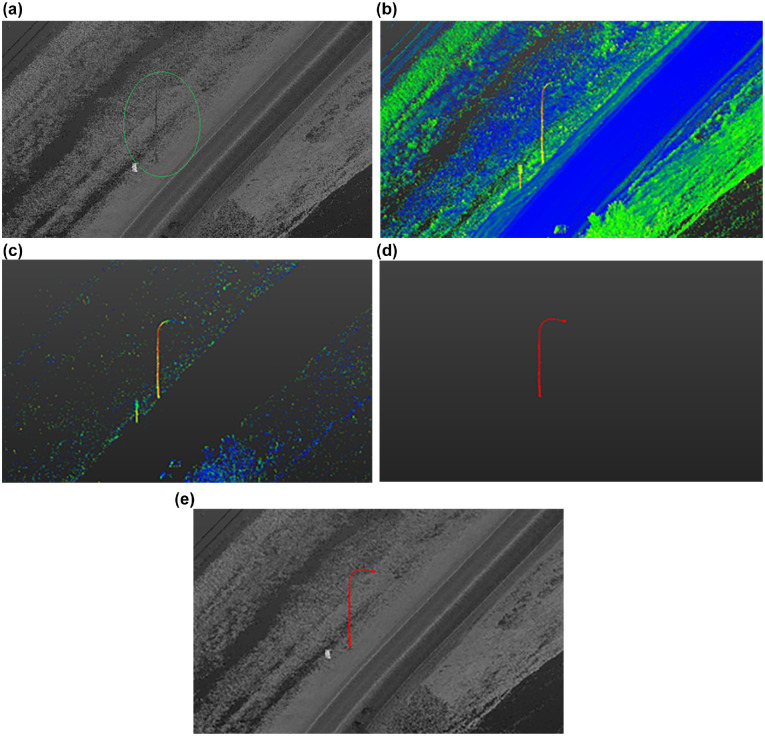
Output visualization of the proposed method: (*a*) light pole on Highway 1, (*b*) z-gradient visualization, (*c*) high z-gradient points extraction, (*d*) surface density filtration, automated clustering, and removal of clusters with low number of points, and (*e*) visualization of final pole points detected.

Each 4 km point cloud is processed separately to reduce processing time. First, the vehicle trajectory is extracted using scan angle and then a method is used to cut the data into 50 m segments for individual analysis. Ground points are filtered based on z-gradient and signs/trees are filtered using surface density. Segments are then rejoined, and poles are isolated based on geometric filtering. Finally, pole location data are exported as comma separated values (CSV) and keyhole markup language (KML) files for further analysis and used in ArcGIS to find collisions at the location. Figure 3 shows a flowchart of the proposed method. Three distinct stages are applied: data preprocessing, light pole filtering, and extraction. Data preprocessing takes the input LAS file and segregates it into individual sections to reduce memory requirements. In filtration, extraneous points belonging to structures such as the ground, trees, and signs are removed, leaving only points belonging to poles in the remaining data. Finally, extraction involves the identification of individual pole objects and documentation of pole locations and dimensions, as well as validation of the accuracy of the overall method. Figure 4 shows a visualization of the output of the different steps in the proposed method.

## Trajectory Extraction

**Figure 5.**
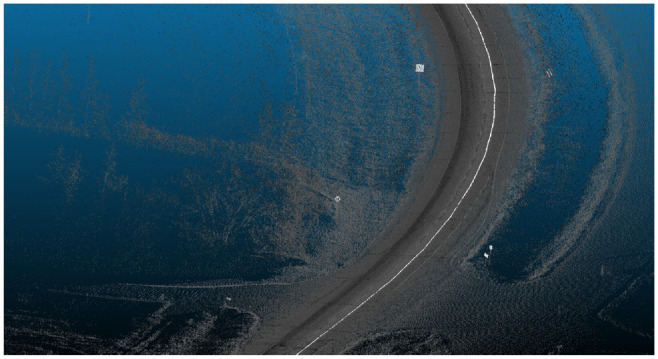
Example of trajectory line.

The scanning vehicle trajectory is determined based on data points located right beneath the scanners and has a scan angle of zero, with each LiDAR scanner creating an individual line of points. Point pairs from each line with equal GPS time are averaged to create a single line centered on the travel lane. An example trajectory line can be seen in Figure 5, and the line calculation is presented in Equation 1.

(1) $$\begin{array}{c} \mathrm{Line}\;\mathrm{Points}=\left\{\frac{S1_{i}+S2_{i}}{2}|\left(\mathrm{SA}\left(S1_{i}\right)=\mathrm{SA}\left(S2_{j}\right)=0\right)\wedge \left(T\left(S1_{i}\right)=T\left(S2_{j}\right)\right)\right\}\; \end{array}$$

where SA(x) is scan angle, T(x) is GPS time, *S*1 is the set of scanner 1 trajectory points, S2 is the set of scanner 2 trajectory points, and *i* and *j* are indices for points in the sets *S1* and *S2*, respectively.

## Extraneous Points Removal

Before filtering, the input LAS file is segmented into 50 m sections because of memory constraints. A 1 m overlap is included between segments both to ensure poles are entirely contained within a single segment and to aid the realignment of neighboring segments after filtration is completed. This is done by selecting start and end points for the segments spaced 50 m apart on the trajectory line. 2D normal vectors are then calculated based on the neighboring trajectory points and traversed a preset distance in both directions laterally to define the corner points of the segment. Segment points are then identified as points within the quadrilateral bounded by the corner points. Road points are initially filtered out based on low z-gradient values, leaving only the surrounding roadside for analysis. Z-gradient is calculated as

(2) $$\begin{array}{c} \vec{\nabla }f_{p}=\frac{1}{k}\sum_{i=1}^{k}\frac{\vec{PP_{i}}}{{\left\|\vec{PP_{i}}\right\|}^{2}}(f(P_{i})-f\left(P\right)) \end{array}$$

where f(x) is the z value of point x , P is the given point to calculate gradient for, and $P_{i}$ are points in a set of k neighbor points evenly sampled in a radius around the given point (*36*).

Additional points are removed based on low surface density; this filters out pole-like objects such as trees. Defining S as the filtered non-ground points, this operation is shown by

(3) $$\begin{array}{c} M=\left\{p\in S|\frac{N\left(p\right)}{\pi R^{2}}>T\right\} \end{array}$$

where R is the surface search radius, T is the threshold surface density (∼15 points/m^2^), M is the final set of filtered points, and N(p) is the number of neighbor points within the search radius around p .

Filtration thresholds for both z -gradient and surface density are manually configurable based on input file properties. Outlying points are then removed using statistical outlier removal (SOR) (*37*). This process is defined in Equations 4–7 below.

For each point $m_{i}\in M$ , the average squared Euclidean distance $\bar{d_{i}}$ to its k nearest neighbor points is first computed as defined in Equation 4. Each point $m_{i}$ is then added to a local cluster of points $C_{L}\in C$ based on proximity, where C is the set of all point clusters.

(4) $$\begin{array}{c} \bar{d_{i}}=\frac{1}{k}\sum_{i}^{k}\mathrm{Ndist}\left(k,\;m_{i}\right)\; \end{array}$$

where $\mathrm{Ndist}(k,\;m_{i})$ is the squared Euclidean distance of the k th closest nearest neighbor to $m_{i}$ .

Additional information is then calculated for use in outlier removal. This includes the average nearest neighbor distance for all points ( μ ) (Equation 5), the standard deviation of nearest neighbor distances ( ξ ) (Equation 6), and the total number of points in all used clusters ( $\mu_{u}$ ) (Equation 7).

(5) $$\begin{array}{c} \mu =\frac{1}{M_{p}}\sum_{i}^{M_{p}}d_{i}\; \end{array}$$

(6) $$\begin{array}{c} \xi =\sqrt{\frac{1}{M_{p}}\sum_{i}^{M_{p}}{\left(d_{i}-\mu \right)}^{2}} \end{array}$$

(7) $$\begin{array}{c} \mu_{u}=\frac{1}{U}\sum_{0}^{M_{p}}\mathrm{NumberPoints}\left(C_{u}\right) \end{array}$$

where $C_{u}$ are used clusters and *U* is the number of used clusters.

Points are then filtered out, which have farther distances than a preset number ( α ) of standard deviations away from the mean distance, as shown in

(8) $$\begin{array}{c} \mathrm{Final}\;\mathrm{Points}=\left\{m_{i}\epsilon M|\left(\mu -\alpha \xi \right)\leq \bar{d_{i}}\leq \left(\mu +\alpha \xi \right)\right\} \end{array}$$

**Figure 6.**
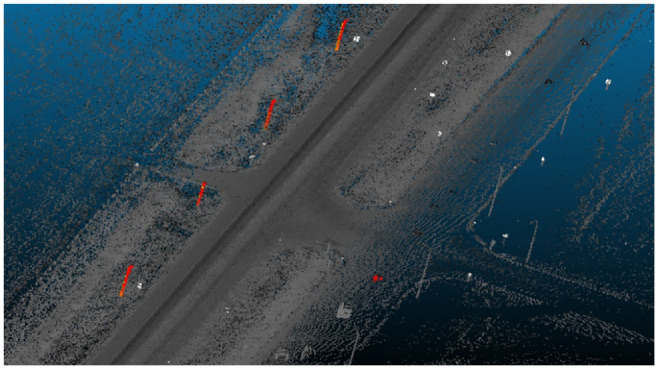
Filtered point cloud.

The individual 50 m sections are then reconnected to create a single point cloud. An example of the reconnected cloud can be seen in Figure 6.

## Pole Identification

From the filtered cloud, individual poles are identified by grouping nearby points into clusters using density-based spatial clustering of applications with noise (DBSCAN) (*38*). DBSCAN groups points based on proximity and hit count. Hit count is defined as the number of points in a cluster. The proximity variable constrains the distance between groups of points that allows them to be considered as one cluster. Therefore, small groups of points or points with long distances between each other are not considered as one cluster. We defined appropriate values for proximity and hit count that produced the best results.

In this study, DBSCAN functions by testing each point for the number of neighboring points within a preset radius of 1.0 m. If the number of points exceeds a threshold value of 17, that group is considered a cluster. Each point in the cluster is recursively searched for more neighbors, adding those points to the cluster until all connected points are grouped. This process is repeated until all points are either considered as part of a cluster or discarded as extraneous noise. This was achieved by modifying the DBSCAN clustering algorithm to operate on 3D data rather than its conventional 2D nature.

**Figure 7.**
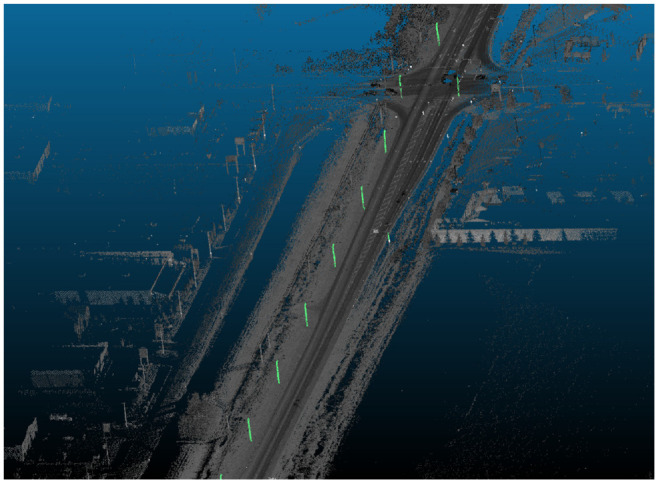
Point clusters.

Each cluster is finally interpreted as a single pole and verified based on dimensions. For each cluster, its centroid is calculated and compared with height standards defined by Alberta Transportation. Any clusters that do not fall within height specifications are then discarded. The remaining pole location and height data are then exported as CSV and KML files. Figure 7 shows a sample of the output on Highway 20.

## Results

**Figure 8.**
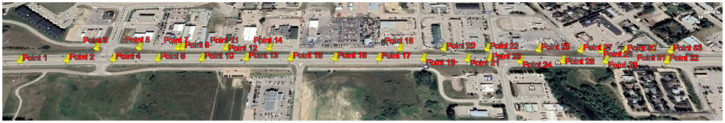
Pole locations in Google Maps.

**Figure 9.**
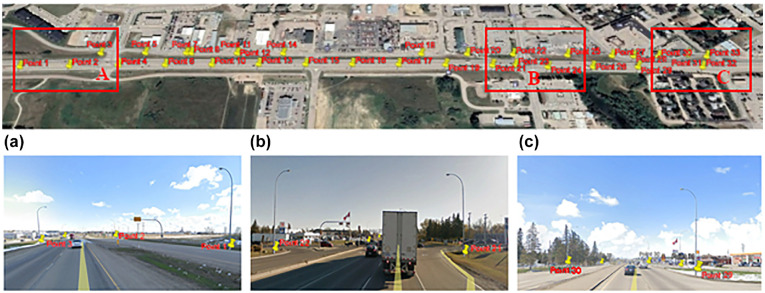
Street view of pole locations at sections A, B, and C.

Table 1 shows all validation results on the seven tested segments. Validation of pole locations results was done by importing calculated pole coordinates into Google Maps. Example outputs from a 4 km Highway 1 segment are shown in Figures 8 and 9.

**Table 1.** Pole Detection Accuracies

Sample name	True positive	False positive	False negative	Precision (%)	Recall (%)	F1 score (%)
Highway 1—Segment 1	33	0	2	100.00	94.29	97.06
Highway 1—Segment 2	5	0	0	100.00	100.00	100.00
Highway 1—Segment 3	7	0	0	100.00	100.00	100.00
Highway 1—Segment 4	5	0	0	100.00	100.00	100.00
Highway 1—Segment 5	6	0	0	100.00	100.00	100.00
Highway 20	42	2	0	95.45	100.00	97.67
Highway 11	6	0	0	100.00	100.00	100.00

Quantitative evaluation of the proposed method was done based on three metrics: precision, recall, and F1 scores as shown in Equations 9 to 11. Comparison was then made to existing state-of-the-art work done by Gargoum et al. (*19*) on light pole mapping, as can be seen in Table 2.

**Table 2.** Comparison with Existing Methods

Matrices	Proposed method (%)	Gargoum et al. (*19*) (%)	Yang et al. (*26*) (%)	Wu et al. (*30*) (%)	Ha et al. (*32*) (%)	Yan et al. (*33*) (%)
Precision	98.10	68.00	94.56	99.4	95.8	94.1
Recall	98.10	49.00	96.66	98.8	92.9	97.8
F1 score	98.10	56.96	95.60	98.6	94.3	95.9

(9) $$\mathrm{Precision}=\frac{\mathrm{TP}}{\mathrm{TP}+\mathrm{FP}}$$

(10) $$\mathrm{Recall}=\frac{\mathrm{TP}}{\mathrm{TP}+\mathrm{FN}}$$

(11) $$F1=2\;\cdot \;\frac{\mathrm{Precision}\;\cdot \;\mathrm{Recall}}{\mathrm{Precision}+\mathrm{Recall}}$$

where TP, FP, and FN represent true positives, false positives, and false negatives, respectively. True positives are poles that were correctly detected by the algorithm. True negatives are non-pole points removed during the filtering process. False positives are point clusters that were incorrectly classified as poles. False negatives are poles that were not detected by the program.

## Discussion

As evident from Table 1, the proposed algorithm shows accurate results, with overall precision, recall, and F1 scores of 98.1%. The lower precision and F1 scores for highway 20 were caused by a powerline running parallel to the road leading to detections of light pole-like objects. Reducing the buffer zone width on this segment could improve performance. Lower recall and F1 scores in Highway 1 Segment 1 was caused by roadside obstructions blocking sections of poles, causing their average height to differ from design standards and remain unclassified by the program, a limitation similar to those reported in previous studies (*19, 39*). As shown in Table 2, the proposed method also performs well compared with existing work, especially on rural scans (*19, 33*). It is worth mentioning that the research done by Gargoum et al. (*19*) was on the same 4 km segment of rural Highway 20 used in this study. Studies (*26, 30*, *32*) in Table 2 are performed on urban road segments that typically have higher quality scans. Noteworthy is that ground removal methods used in urban research were not successful when applied to our rural data. This was also a challenge and limitation in Gargoum et al. (*19*). The point-based technique proposed showed significantly better performance and is more suitable to rural environments. The total number of points in the data set is 125,540,804 points. The processing time of a 4 km segment with 21 million points is approximately 3 to 4 min.

Heavy vegetation surrounding poles can pose a challenge to the extraction process. In this situation, the difference in geometric properties between the vegetation and pole points presents an opportunity for further filtering. Testing our method on an electric pole covered by heavy vegetation, a density-filter was applied to the point cloud after the extraction of high z-gradient points. This modification to the algorithm produced favorable results.

The above procedure can be used to explore the relationship between traffic collisions and the mapped pole locations in a geographic information system (GIS) platform. This would facilitate in-depth safety assessments similar to the earlier work by El Esawey et al. (*1*). Their work showed the importance of adequate clear zones required by design guidelines to provide a more forgiving environment on highways (*1*). Unfortunately, the authors have pointed out the difficulties and challenges they faced in extracting the poles’ locations, which are now easily addressed using the proposed extraction procedure.

Transportation agencies base their road design guidelines on empirical values, a fact that has stimulated research in the optimization of these guidelines. Poles have proven to be a frequent cause of crashes on roadways, so a model that tracks which pole locations are more dangerous than others can help transportation agencies make informed decisions on this aspect of road design. Furthermore, because of the versatility of LiDAR, many other geographical features relating to pole locations, such as ground slope or distance from travel lane, can be extracted synchronously. In the event of a pole-related collision, a police officer is responsible for officially reporting the cause and location. With the coordinates of the crash site, transportation agencies must manually go to the location to note which light pole was the cause, along with many other details that may reveal an underlying theme with light pole crashes. Overall, this is a time-consuming and laborious task with the potential to be streamlined with automation. The proposed method is capable of assisting with this very problem, allowing for virtually every light pole and its parameters to be extracted and overlapped with crash history and the properties of those crashes (e.g., severity, etc.) in a GIS platform. With all this information about light poles readily available, transportation agencies could easily perform roadside assessment and safety audits to create a performance-based light pole placement guideline. Currently, according to Alberta Transportation’s Highway Lighting Guide, “standard pole setback in rural areas shall be 5.0 m from the edge of the through lane (white line) to the face of the lighting pole” unless protected by a barrier. That being said, there exist a few exceptions to this rule where deemed appropriate (e.g., high crash rates). To enhance roadside safety, the proposed method, given that it allows for recording all light pole dimensions and positions, can help determine where further exceptions can be allowed, such as further setback, light pole redesign, or light pole removal. The method can also help build training data sets that could be used to develop deep learning segmentation models.

## Conclusions and Future Work

This paper proposes an automated approach to detect and map light poles on rural highway segments scanned at high speeds. By extracting and smoothing the scanning vehicle’s trajectory, the point-cloud data are then segmented into smaller overlapped tiles of data. To extract pole objects, several filters based on z-gradient values, density, and statistical outlier removal are applied. The segmented data sections are then recombined, and a density-based clustering algorithm is used to group the remaining points into clusters. A geometric filter is finally applied to extract poles based on their dimensions. The proposed algorithm shows promising results relative to previous studies, with an average precision, recall, and F1 scores reaching 98.1% for the tested segments. When the model was applied on a pole-like object covered by heavy vegetation, it successfully extracted pole points and their location with the addition of a density-based filter on detected high z-gradient points. The proposed approach can help transportation agencies build light pole inventories on a network level and automate related road safety audits. One limitation of the study is that poles that were occluded by other objects during scanning may not be extracted. Further research may explore solving this limitation using point-based deep learning and data augmentation approaches (*34, 35, 40*). It is suggested that future studies apply a similar approach on a larger sample size and use the collected data to calibrate safety performance functions that model the relationship between pole collision frequency and roadside variables, as suggested by El Esawey and Sayed (*1*), Gouda et al. (*16*). The relationship between point-cloud density and light pole extraction is worth further investigation. Finally, studying the relationship between lighting conditions and nighttime collisions is suggested.

## References

[1] El Esawey M. Sayed T. Evaluating Safety Risk of Locating Above Ground Utility Structures in the Highway Right-of-Way. Accident Analysis & Prevention, Vol. 49, 2012, pp. 419–428. 10.1016/j.aap.2012.03.008 23036421

[2] NHTSA. Fatality Analysis Reporting System (FARS), 2020. https://www.nhtsa.gov/research-data/fatality-analysis-reporting-system-fars.

[3] Gargoum S. A. El-Basyouny K. Shalkamy A. Gouda M. Feasibility of Extracting Highway Vertical Profiles from LiDAR Data. Canadian Journal of Civil Engineering, Vol. 45, No. 5, 2018, pp. 418–421.

[4] Gouda M. Mirza J. Weiß J. Castro A. R. El-Basyouny K. Octree-Based Point Cloud Simulation to Assess the Readiness of Highway Infrastructure for Autonomous Vehicles. Journal of Computer-Aided Civil and Infrastructure Engineering, Vol. 36, 2021, pp. 922–940.

[5] Shalkamy A. El-Basyouny K. Xu H. Y. Voxel-Based Methodology for Automated 3D Sight Distance Assessment on Highways Using Mobile Light Detection and Ranging Data. Transportation Research Record: Journal of Transportation Research Board, 2020. 2674: 587–599.

[6] Shalkamy A. Karsten L. Gargoum S. El-Basyouny K. A Framework to Detect Horizontal Curves and Assess Their Geometric Properties from Remotely Sensed Point Clouds. International Journal of Remote Sensing, Vol. 41, No. 21, 2020, pp. 8328–8351.

[7] Shalkamy A. El-Basyouny K. Multivariate Models to Investigate the Relationship between Collision Risk and Reliability Outcomes on Horizontal Curves. Accident Analysis & Prevention, Vol. 147, 2020, p. 105745. 10.1016/j.aap.2020.105745 32947175

[8] Habib K. Gouda M. El-Basyouny K. Calibrating Design Guidelines Using Mental Workload and Reliability Analysis. Transportation Research Record: Journal of Transportation Research Board, 2020. 2674: 360–369.

[9] Agina S. Shalkamy A. Gouda M. El-Basyouny K. Automated Assessment of Passing Sight Distance on Rural Highways Using Mobile LiDAR Data. Transportation Research Record: Journal of Transportation Research Board, 2021. 2675: 676–688.

[10] Shalkamy A. Gargoum S. El-Basyouny K. Towards a More Inclusive and Safe Design of Horizontal Curves: Exploring the Association between Curve Features, Reliability Measures, and Safety. Accident Analysis & Prevention, Vol. 153, 2021, p. 106009. 10.1016/j.aap.2021.106009 33581606

[11] Cabo C. Ordoñez C. García-Cortés S. Martínez J. An Algorithm for Automatic Detection of Pole-Like Street Furniture Objects from Mobile Laser Scanner Point Clouds. ISPRS Journal of Photogrammetry and Remote Sensing, Vol. 87, 2014, pp. 47–56.

[12] Gargoum S. A. El-Basyouny K. A Literature Synthesis of LiDAR Applications in Transportation: Feature Extraction and Geometric Assessments of Highways. GIScience & Remote Sensing, Vol. 56, No. 6, 2019, pp. 864–893.

[13] Gouda M. Chowdhury I. Weiß J. Epp A. El-Basyouny K. Automated Assessment of Infrastructure Preparedness for Autonomous Vehicles. Automation in Construction, Vol. 129, 2021, p. 103820.

[14] Gouda M. Mello B. Visser J. El-Basyouny K. Eliminating Infrastructure Barriers for Autonomous Driving: A Case of Vertical Curve Design. Presented at 100th Annual Meeting of the Transportation Research Board, Washington, D.C., 2021.

[15] Gargoum S. El-Basyouny K. Effects of LiDAR Point Density on Extraction of Traffic Signs: A Sensitivity Study. Transportation Research Record: Journal of Transportation Research Board, 2019. 2673: 41–51.

[16] Gouda M. Mello B. El-Basyouny K. Automated Safety Assessment of Roadside Clear Zones Using LiDAR Data. Transportation Research Record: The Journal of the Transportation Research Board, 2021. 2675: 432–448. 10.1177/03611981211029934 PMC13038139 41924353

[17] Kilani O. Gouda M. Weiß J. El K.-Basyouny. Safety Assessment of Urban Intersection Sight Distance Using Mobile LiDAR Data. Sustainability, Vol. 13, No. 16, 2021, p. 9259.

[18] Yan W. Y. Morsy S. Shaker A. Tulloch M. Automatic Extraction of Highway Light Poles and Towers from Mobile LiDAR Data. Optics & Laser Technology, Vol. 77, 2016, pp. 162–168.

[19] Gargoum S. A. James Koch C. El-Basyouny K. A Voxel-Based Method for Automated Detection and Mapping of Light Poles on Rural Highways Using LiDAR Data. Transportation Research Record: The Journal of the Transportation Research Board, 2018. 2672: 274–283.

[20] Lehtomäki M. Jaakkola A. Hyyppä J. Kukko A. Kaartinen H. Detection of Vertical Pole-Like Objects in a Road Environment Using Vehicle-Based Laser Scanning Data. Remote Sensing, Vol. 2, No. 3, 2010, pp. 641–664.

[21] Yokoyama H. Date H. Kanai S. Takeda H. Pole-Like Objects Recognition from Mobile Laser Scanning Data Using Smoothing and Principal Component Analysis. International Archives of the Photogrammetry, Remote Sensing and Spatial Information Sciences, Vol. 38, 2011, pp. 115–120.

[22] Golovinskiy A. Kim V. G. Funkhouser T. Shape-Based Recognition of 3D Point Clouds in Urban Environments. Proc., 2009 IEEE 12th International Conference on Computer Vision, Kyoto, Japan, IEEE, New York, 2009, pp. 2154–2161.

[23] Pu S. Rutzinger M. Vosselman G. Elberink S. O. Recognizing Basic Structures from Mobile Laser Scanning Data for Road Inventory Studies. ISPRS Journal of Photogrammetry and Remote Sensing, Vol. 66, No. 6, 2011, pp. S28–S39.

[24] Yu Y. Li J. Guan H. Wang C. Yu J. Semiautomated Extraction of Street Light Poles from Mobile LiDAR Point-Clouds. IEEE Transactions on Geoscience and Remote Sensing, Vol. 53, No. 3, 2014, pp. 1373–1386.

[25] Zheng H. Tan F. Wang R. Pole-Like Object Extraction from Mobile LiDAR Data. International Archives of the Photogrammetry, Remote Sensing and Spatial Information Sciences, Prague, Czech Republic, 2016.

[26] Yang J. Kang Z. Akwensi P. H. A Skeleton-Based Hierarchical Method for Detecting 3-D Pole-Like Objects from Mobile LiDAR Point Clouds. IEEE Geoscience and Remote Sensing Letters, Vol. 16, No. 5, 2019, pp. 801–805.

[27] Wu B. Yu B. Yue W. Shu S. Tan W. Hu C. Huang Y. Wu J. Liu H. A Voxel-Based Method for Automated Identification and Morphological Parameters Estimation of Individual Street Trees from Mobile Laser Scanning Data. Remote Sensing, Vol. 5, No. 2, 2013, pp. 584–611.

[28] Tu J. Yao J. Li L. Zhao W. Xiang B. Extraction of Street Pole-Like Objects Based on Plane Filtering from Mobile LiDAR Data. IEEE Transactions on Geoscience and Remote Sensing, Vol. 59, No. 1, 2021, pp. 749–768.

[29] Lehtomäki M. Jaakkola A. Hyyppä J. Lampinen J. Kaartinen H. Kukko A. Puttonen E. Hyyppä H. Object Classification and Recognition from Mobile Laser Scanning Point Clouds in a Road Environment. IEEE Transactions on Geoscience and Remote Sensing, Vol. 54, No. 2, 2016, pp. 1226–1239.

[30] Wu F. Wen C. Guo Y. Wang J. Yu Y. Wang C. Li J. Rapid Localization and Extraction of Street Light Poles in Mobile LiDAR Point Clouds: A Supervoxel-Based Approach. IEEE Transactions on Intelligent Transportation Systems, Vol. 18, No. 2, 2016, pp. 292–305.

[31] Guan H. Yu Y. Li J. Liu P. Pole-Like Road Object Detection in Mobile LiDAR Data Via Supervoxel and Bag-of-Contextual-Visual-Words Representation. IEEE Geoscience and Remote Sensing Letters, Vol. 13, No. 4, 2016, pp. 520–524.

[32] Thanh Ha T. Chaisomphob T. Automated Localization and Classification of Expressway Pole-Like Road Facilities from Mobile Laser Scanning Data. Advances in Civil Engineering, Vol. 2020, 2020, p. 5016783.

[33] Yan L. Li Z. Liu H. Tan J. Zhao S. Chen C. Detection and Classification of Pole-Like Road Objects from Mobile LiDAR Data in Motorway Environment. Optics & Laser Technology, Vol. 97, 2017, pp. 272–283.

[34] Qi C. R. Su H. Mo K. Guibas L. J. Pointnet: Deep Learning on Point Sets for 3D Classification and Segmentation. Proc., IEEE Conference on Computer Vision and Pattern Recognition, San Francisco, CA, 2017, pp. 652–660.

[35] Gouda M. El-Basyouny K. Epp A. Traffic Sign Extraction using Deep Hierarchical Feature Learning and LiDAR Data on Rural Highways. Presented at the 100th Annual Meeting of Transportation Research Board, Washington, D.C., 2021.

[36] Girardeau-Montaut D. Détection de changement sur des données géométriques tridimensionnelles. Doctoral dissertation. Telecom Paris, 2006.

[37] Balta H. Velagic J. Bosschaerts W. De Cubber G. Siciliano B. Fast Statistical Outlier Removal Based Method for Large 3D Point Clouds of Outdoor Environments. IFAC-PapersOnLine, Vol. 51, No. 22, 2018, pp. 348–353.

[38] Ester M. Kriegel H. -P. Sander J. Xu X. A Density-Based Algorithm for Discovering Clusters in Large Spatial Databases with Noise. KDD, Vol. 96, No. 34, 1996, pp. 226–231.

[39] El-Halawany S. I. Lichti D. D. Detecting Road Poles from Mobile Terrestrial Laser Scanning Data. GIScience & Remote Sensing, Vol. 50, No. 6, 2013, pp. 704–722.

[40] Gouda M. El-Basyouny K. Epp A. A Deep Learning Algorithm to Extract Traffic Signs from Point Cloud Data. Presented at 99th Annual Meeting of the Transportation Research Board, Washington, DC, 2020.

